# Enhanced Alarmin Secretion Exacerbates Neutrophil Extracellular Trap (NET) Formation in Active Psoriasis: Implication of IL-33 and TSLP in Driving NET Formation, Inflammation and Oxidative Stress in Psoriasis

**DOI:** 10.3390/antiox15010071

**Published:** 2026-01-06

**Authors:** Vanshika Ojha, Manoj Kumar Tembhre, Vishal Gupta

**Affiliations:** 1 Department of Cardiac Biochemistry, All India Institute of Medical Sciences (AIIMS), New Delhi 110029, India; 2 Department of Dermatology and Venereology, All India Institute of Medical Sciences (AIIMS), New Delhi 110029, India

**Keywords:** psoriasis, alarmins, neutrophil extracellular traps (NETs), oxidative stress, keratinocyte proliferation

## Abstract

Psoriasis is a common inflammatory skin disease with chronic manifestation in which the role of neutrophil extracellular traps (NETs) and alarmins are increasingly recognized as contributors to systemic and cutaneous inflammation. However, the interaction between alarmins and NET-driven immune responses remains poorly defined. The main aim of this study is to define the role of target alarmins (i.e., IL-33 and TSLP) in NETs induction and its subsequent impact on oxidative stress and inflammation in the peripheral blood. In the present study, we recruited active psoriasis patients (*n* = 56) and control (*n* = 56) subjects. The frequency of circulating neutrophils, the levels of NET-associated markers (MPO (myeloperoxidase)–DNA complex, CitH3 (citrullinated histone H3), PAD4 (peptidyl arginine deiminase4), NADPH oxidase, and NE (neutrophil elastase)), and alarmin transcripts (IL (interleukin)-33, TSLP (thymic stromal lymphopoietin), S100A7, S100B, HSP (heat shock protein) 60/70 were quantified using flow cytometry, ELISA (Enzyme-linked immunosorbent assay), and qPCR (quantitative polymerase chain reaction), respectively, in each group. The NET formation potential of isolated neutrophils was assessed in the presence or absence of rhIL-33 and rhTSLP by immunocytofluorescence. The effect of rhIL-33- and rhTSLP-primed NETs in augmenting oxidative stress and inflammation was evaluated on peripheral blood mononuclear cells (PBMCs) by ELISA. Significantly higher circulating neutrophils (*p* < 0.001) and levels of NET-associated markers (i.e., MPO–DNA complex, CitH3, PAD4, NADPH oxidase, and NE) were observed in active psoriasis patients compared to controls. Lesional skin exhibited strong expression of MPO (*p* < 0.001) compared to normal skin. The alarmins, IL-33 and TSLP, were markedly upregulated in the blood and skin (*p* < 0.05). The rhIL-33 and rhTSLP treated neutrophils demonstrated enhanced NETosis in patients (*p* < 0.001). Increased expression of inflammatory cytokines and oxidative stress markers were reported in PBMCs when incubated with rhIL-33- and rhTSLP-primed NETs. Taken together, our investigation demonstrated the novel mechanism wherein the alarmins IL-33 and TSLP exacerbate NET formation that may drive enhanced inflammation and oxidative stress in psoriasis.

## 1. Introduction

Psoriasis is a common inflammatory skin disease often presented as a chronic manifestation affecting nearly 2–3% of individuals globally. Psoriasis is recognized by excessive keratinocyte proliferation and impaired differentiation, with an infiltration of immune cells leading to epidermal thickening and the formation of scaly plaques on the skin [1,2]. Psoriasis has a multifactorial etiology involving immunological, genetic, and environmental factors. Over the last decade, the IL (interleukin)-23/IL-17 axis has been recognized as the critical driver of inflammation in psoriasis shaping both disease initiation and maintenance [3,4]. However, psoriasis is not solely an adaptive immune disorder [5,6,7]. A growing body of evidence highlights that the earliest triggers of disease lie within dysregulated innate immune responses, particularly those initiated by epithelial- and immune-derived alarmins [8,9]. These molecules are released from stressed keratinocytes, activated immune cells, and damaged tissues serving as endogenous danger signals and activating the immune system [10]. Among them, the S100 family proteins [11,12], S100A7 [13], S100B [14], IL-33 [15], TSLP (thymic stromal lymphopoietin) [16,17], and HSP (heat shock protein) 60 and HSP70 [18,19] have emerged as the key modulators of inflammation. In healthy conditions, alarmins perform intracellular housekeeping roles [9]; however, upon their extracellular release, they bind to innate immune receptors, such as TLRs (toll-like receptors) and RAGE (receptor for advanced glycation end products), triggering potent inflammatory cascades [7]. Several alarmins are consistently elevated in psoriatic skin and serum, correlating with disease activity and contributing to keratinocyte activation, angiogenesis, and immune cell recruitment [20]. Further, neutrophils are abundant in psoriatic lesions [21,22], where they accumulate in the epidermis to form Munro microabscesses and contribute to tissue damage through the secretion of proteases, cytokines, and reactive oxygen species (ROS) [23]. Besides their phagocytic role, one of their most important effector mechanisms is neutrophil extracellular trap (NET) formation, a specialized form of cell death (i.e., NETosis) in which neutrophils release chromatin enriched with enzymes such as MPO (myeloperoxidase), NE (neutrophil elastase), proteinase 3, and various other inflammatory proteins (e.g., CitH3 (citrullinated histone 3), cathepsin, calprotectin, etc.) [24]. NETs are increasingly recognized as pathogenic amplifiers in various infectious, autoimmune, and inflammatory diseases, including psoriasis [25,26,27,28]. The NETs armamentarium consists of potent inflammatory molecules that can activate the type I interferon secreting pDCs (plasmacytoid dendritic cells) that promote IL-23 release, enhance Th17 responses, and stimulate keratinocyte proliferation [28,29]. Elevated NET-associated markers, such as MPO–DNA complexes and circulating CitH3, PAD4 (peptidyl arginine deiminase4), and NE levels, have been consistently reported in psoriasis patients, suggesting the chronic elevation of NETosis in the disease [30,31,32]. Despite these observations, the precise mechanistic contribution of alarmins towards neutrophil activation and downstream effector pathways remains incompletely defined. Yet, a major unanswered question is what drives the heightened NETotic potential of neutrophils in psoriasis, and alarmins are likely an upstream trigger. Since neutrophils respond strongly to DAMPs (damage-associated molecular patterns), alarmins offer an interesting but understudied upstream link between neutrophil dysregulation and epithelial injury. Parallel to immune dysregulation, oxidative stress [33,34,35] represents another crucial but poorly understood feature of psoriasis pathobiology. High levels of lipid peroxidation, DNA damage, and protein oxidation, along with reduced antioxidant mechanisms mediated by catalase, superoxide dismutase, and glutathione has been reported in psoriasis [34,35,36,37]. Oxidative stress not only contributes to keratinocyte hyperproliferation and inflammatory cytokine release but serves as a key mediator of NETosis [38]. The combined effects of alarmin dysregulation, neutrophil activation and redox imbalance suggest the existence of a feedforward inflammatory loop in psoriasis that is still not completely defined. Despite many insights into adaptive immunity, our understanding of the innate immune events that precede and drive these adaptive immune responses in psoriasis is still incomplete. Considering the mentioned gaps, we aim to define the role of NETs and their associated markers (MPO–DNA complexes, CitH3, PAD4, NE, and NADPH oxidase activity) in peripheral blood and lesional skin in relation to alarmins. Further, the role of alarmin-primed NETs in eliciting the inflammatory cytokine response (i.e., IFN (Interferon)-γ), IL-6, IL-15, IL-17, TNF (tumor necrosis factor)-α, IL-13, IL-23) and oxidative stress markers (i.e., MDA (malondialdehyde), 8-OHdG (8-hydroxy-2′-deoxyguanosine), SOD (superoxide dismutase), GSH/GSSG (glutathione/glutathione disulfide), and catalase) was evaluated in active psoriasis patients. This is the first study establishing the role of alarmins (IL-33/TSLP) in inducing NETs and its subsequent impact in triggering oxidative stress and inflammation in the peripheral blood of psoriasis patients demonstrating a novel IL-33–TSLP–NETs triad axis involvement in the pathogenesis of psoriasis.

## 2. Materials and Methods

### 2.1. Study Subjects and Sampling

A total of 112 participants were recruited from the Dermatology Outpatient Department (OPD) at AIIMS, New Delhi. Only patients with plaque-type psoriasis, i.e., psoriasis vulgaris, were included in the patient group. Individuals with psoriatic arthritis (PsA) were systematically excluded based on clinical evaluation and radiological history. Disease severity was assessed using the Psoriasis Area and Severity Index (PASI). Eligible patients had active psoriasis, were treatment-naïve, or had not received systemic or topical therapy (including corticosteroids, retinoids, phototherapy, methotrexate, cyclosporine, or biologics) for four weeks preceding sample collection. To minimize any interfering variable, the following were excluded in both groups: metabolic syndrome (e.g., obesity, dyslipidemia), cardiovascular diseases, autoimmune diseases (autoimmune thyroiditis, rheumatoid arthritis (RA), inflammatory bowel disease (IBD), systemic lupus erythematosus (SLE)), associated skin diseases (vitiligo, PsA, alopecia areata, acne, dermatitis, etc.), systemic infections, recent immunotherapy, smoking, alcohol or any drug addiction, and a BMI ≥ 25. The study population comprised 56 active psoriasis patients (*n* = 47 males, *n* = 9 females) and 56 age-matched healthy controls (*n* = 39 males, *n* = 17 females). Demographic details are presented in Table 1. Blood samples were collected from all participants, and 3–4 mm skin biopsies were obtained from the lesional skin of active psoriasis patients. For comparison, normal skin samples (*n* = 56) were obtained from healthy subjects undergoing scar revision or circumcision procedures. All the patients and control subjects did not have any associated inflammatory, autoimmune, or infectious disease at the time of study enrolment. Written informed consent was obtained from all the participants. The study protocol was approved by the Institutional Ethics Committee of AIIMS, New Delhi, and the study was conducted in accordance with the declaration of Helsinki for human studies.

### 2.2. PBMC Isolation

Peripheral blood samples were collected from active psoriasis patients and healthy controls. PBMCs were isolated using Histopaque^®^-1077 (Sigma-Aldrich, St. Louis, MO, USA) density gradient centrifugation as per the manufacturer’s instructions. The isolated PBMC layer was carefully aspirated, washed twice with sterile phosphate-buffered saline (PBS), and resuspended in a complete RPMI-1640 medium (Sigma-Aldrich, St. Louis, MO, USA) having 10% fetal bovine serum (FBS) (Thermo Fisher Scientific, Carlsbad, CA, USA), and was used for further analysis as described in subsequent sections.

### 2.3. Neutrophil Isolation, NET Formation, and Stimulation Assay: Identification and Quantification

Neutrophils were isolated from the peripheral blood of active psoriasis patients and healthy controls using a two-step method involving Histopaque^®^-1119-based density gradient centrifugation (Sigma-Aldrich, St. Louis, MO, USA), followed by a Percoll (Sigma-Aldrich, St. Louis, MO, USA) gradient separation. Isolated cells were washed with sterile 1× PBS and seeded onto 24-well culture plates containing 13-mm round glass coverslips in an RPMI-1640 medium (Sigma-Aldrich, St. Louis, MO, USA) containing 2% FBS (Thermo Fisher Scientific, Carlsbad, CA, USA). Neutrophil viability and purity were assessed by multicolor flow cytometry (Supplementary Figure S1) prior to NET induction, as described previously [39]. The cells were then seeded at a concentration of 1 × 10^6^ cells/mL per well and were allowed for adherence for one hour (h). Following adherence, neutrophils were subjected to three different treatments: (i) phorbol 12-myristate 13-acetate (PMA; 600 nM; Sigma-Aldrich, St. Louis, MO, USA) for 4 h at 37 °C (PMA served as a positive control for NET induction, no PMA (only media) was added in the negative control), (ii) recombinant human IL-33 (rhIL-33; 100 ng/mL; Sigma-Aldrich, St. Louis, MO, USA), and (iii) recombinant human TSLP (rhTSLP; 100 ng/mL; Sigma-Aldrich, St. Louis, MO, USA) for 4 h at 37 °C. A dose optimization assay was performed for rhIL-33 and rhTSLP at 0 ng/mL (media only), 50 ng/mL and 100 ng/mL. Based on the findings, 100 ng/mL dose concentrations of rhIL-33 and rhTSLP were selected for all subsequent experiments as maximum response was observed at this dose (Supplementary Figure S2). Subsequently, the rhIL-33- and rhTSLP-primed NETs were then used to stimulate the PBMCs for 24 h followed by RNA extraction and cell lysate preparation to be used for subsequent quantitative polymerase chain reaction (qPCR) analysis and ELISA (Enzyme-linked immunosorbent assay)-based assays, respectively. Additionally, in the similar experimental setup, we performed assays to investigate the direct effect of rhIL-33 and rhTSLP on PBMCs (Supplementary Figure S3). All assays were performed in triplicates. DAPI control was used to rule out the presence of extracellular DNA prior to NET induction. For the quantitative analysis, intact neutrophils were counted at the time of seeding and 1 h after seeding (once cells adhere to the surface prior to stimulation). The glass cover slip was removed from each well and an immunofluorescence-based investigation was performed, as described in a subsequent section. NET formation was assessed using fluorescence microscopy-based image analysis, as mentioned in a subsequent section. Apoptotic/necrotic cells were excluded using a morphological features assessment to distinguish NETosis and NET features (MPO–DNA complexes, decondensed nuclei, extracellular DNA/chromatin). Only cells fulfilling both morphological criteria and MPO–DNA overlap were classified as NETotic. The NET assessment relied on a combined morphological assessment, MPO–DNA colocalization, and mean fluorescent intensity using immunofluorescence microscopy.

### 2.4. RNA Extraction and qPCR Assay

Total RNA was extracted from peripheral blood, skin (lesional and non-lesional) biopsies, and from stimulated PBMCs (as described above) using the RNA extraction kit (Qiagen, Turnberry Lane, Santa Clarita, CA, USA), as per instructions. A nanodrop spectrophotometer (BioTek Epoch 2, Agilent Technologies, Inc., Santa Clara, CA, USA) was used to measure the purity and concentration of the isolated RNA, as described previously [40]. To eliminate any residual DNA contamination, RNA samples were treated with DNase I, as per the manufacturer’s instructions provided in the DNase free kit (Thermo Fisher Scientific, Carlsbad, CA, USA). For complementary DNA (cDNA) synthesis, 1 µg of DNase-treated RNA was reverse transcribed using the iScript™ cDNA Synthesis Kit (Thermo Fisher Scientific, Carlsbad, CA, USA). The resulting cDNA was then used for qPCR analysis. The qPCR was performed using the Maxima SYBR Green qPCR Master Mix (Thermo Fisher Scientific, Carlsbad, CA, USA) in the CFX96 Real-Time PCR System (Bio-Rad, Hercules, CA, USA), according to MIQE guidelines. The standard PCR settings were followed, as described previously [40,41], which includes, briefly, initial denaturation (95 °C; 10 min), followed by 40 cycles of denaturation (95 °C;15 s) with primer annealing (30 s) at the optimized temperature, and final extension (72 °C). All assays were performed in triplicates and, for internal control, beta (β)-actin was used. Mean Ct value was used for analysis and it was normalized against β-actin. Oligonucleotide primers were obtained from Sigma-Aldrich (Table 2). Gene expression data were presented in terms of 2^−ΔCt^ values for both the groups.

**Table 2:** Genes and their forward and reverse oligonucleotide primers.

Genes	Gene Accession Number	Forward Primers (5′-3′) Reverse Primers (3′-5′)
*β-actin*	NM_001101	GCGTGACATTAAGGAGAAG GAAGGAAGGCTGGAAGAG
*IL-33*	NM_033439	GCCAACAACAAGGAACACTCTG CACTCCAGGATCAGTCTTGCAT
*TSLP*	NM_033035	CCAGAGCCTAACCTTCAATCC ATAGCCTGGGCACCAGATAG
*S100A7*	NM_002963	GACTACCACAAGCAGAGCCAT GACATTTTATTGTTCCTGGGGTCT
*S100B*	NM_006272	CATGCAGATAACAGCTGGTTG CGGATTGCGAGTTCTGATGG
*HSP60*	NM_199440	CGCTGACGCGAAGACTCG AGCATTAAGGCTCGGGCATC
*HSP70*	NM_005345	CCACTGGAAGGACTTAGGCG CTGGAAGCCCAGGTCTATGC
*IL-6*	NM_001371096	AGACAGCCACTCACCTCTTC CAGGCTGGCATTTGTGGTTG
*IL-15*	NM_000585	TGGGCCTAGAGTAGCTTACC TTAGGTGCTTTGGGCCAACT
*IL-17*	NM_002190	TGTCCATCTCATAGCAGGCAC CAGGACTCACCACCAATGAG
*IFN-γ*	NM_000619	GCAGAGCCAAATTGTCTCCT ATGCTCTTCGACCTCGAAAC
*TNF-α*	NM_000594	CCATCAGAGGGCCTGTACCT GTGGGTGAGGAGTACATGGG
*IL-13*	NM_002188	ATGCATCCGCTCCTCAATCC TCTGGGTGATGTTGACCAGC
*IL-23*	NM_016584	CCCAAGGACTCAGGGACAAC TGGAGGCTGCGAAGGATTTT

### 2.5. ELISA-Based Assays

Protein quantification/enzyme activity was performed in the serum/cell lysate samples from psoriasis patients and controls using ELISA for MPO–DNA complexes (sensitivity < 10 pg/mL; Abcam, Cambridge, UK), Human CitH3 (sensitivity = 7.04 pg/mL; ELK Biotechnology, Sugar Land, TX, USA), PAD4 (sensitivity = 0.111 ng/mL; Novus Biologicals, Centennial, CO, USA), NADPH oxidase activity (sensitivity = 0.27 U/L; Elabscience Biotechnology Inc., Houston, TX, USA), and NE activity (sensitivity = 0.54 U/L; Elabscience Biotechnology Inc., Houston, TX, USA). In a separate experimental setup, oxidative stress markers were quantified in cell lysates obtained from stimulated PBMCs (as described above). Oxidative stress markers were assessed using colorimetric/fluorometric assay kits for 8-OHdG (sensitivity = 0.59 ng/mL; Abcam, Cambridge, UK), MDA (sensitivity = 0.17 nmol/mL; Elabscience Biotechnology Inc., Houston, TX, USA), SOD (sensitivity = 4.7 U/mL; Elabscience Biotechnology Inc., Houston, TX, USA), catalase activity (sensitivity = 1.12 U/mL; Elabscience Biotechnology Inc., Houston, TX, USA), and GSH/GSSG ratio (sensitivity = 1–100 ng/mL; Abcam, Cambridge, UK). All assays were performed according to the manufacturers’ instructions, as described previously [42]. A microplate reader (Bio-Rad, Hercules, CA, USA) was used to measure the optical density at 450 nm. Standard curves were generated for each assay, and concentrations were expressed in accordance with the specific assay units. Each sample was analyzed in triplicate.

### 2.6. Flow Cytometry-Based Assays

The percentage of neutrophils was estimated in whole blood using the automated Sysmex XN-1000 hematology analyzer (Sysmex India Pvt. Ltd., Mumbai, India) based on the fluorescence flow-cytometry principle. MPO+ neutrophils were estimated in whole blood by fluorescence-tagged MPO antibodies using BD FACSLyric™ (BD Biosciences, Franklin Lakes, NJ, USA), following a standard method. Briefly, 100 µL of EDTA whole blood were lysed with 1× BD FACS™ Lyse Buffer for 10 min at room temperature (RT), followed by centrifugation (400× *g* for 5 min) and washing with 1× PBS. 4% paraformaldehyde was used to fix the cells for 20 min at RT, and were washed and permeabilized (30 min, 4 °C) with BD Perm/Wash™ Buffer. Permeabilized cells were incubated (20 min, at 4 °C in the dark) with FITC-conjugated anti-human MPO antibody (BioLegend, San Diego, CA, USA), washed twice and resuspended in PBS. In addition, the viability and purity of neutrophils were assessed by multicolor flow cytometry prior to NET induction. For the viability assessment, freshly isolated neutrophils were stained with SYTOX™ Green (Invitrogen™, Thermo Fisher Scientific, Carlsbad, CA, USA), a membrane-impermeant nucleic acid dye, according to the manufacturer’s instructions, and immediately acquired on a flow cytometer. For the purity assessment, neutrophils were fixed and stained with surface antibodies for antihuman-CD16-PerCP.Cy5.5 and antihuman-CD14-PECy7, followed by intracellular staining with anti-human-MPO antibody after permeabilization (as explained above). Samples were then acquired on a BD FACSLyric™ (BD Biosciences, USA) and one lakh event was recorded per sample. Doublets were excluded using FSC-A vs. FSC-H gating, followed by the sequential gating of granulocytes. Unstained and isotype controls were used to set the gates, and compensation was applied using BD™ CompBeads. Data were analyzed using BD FACSuite™ v6.0, and cell frequency was expressed as a percentage of the total granulocytes. For cell viability, within the singlet granulocyte gate, viable neutrophils were identified as SYTOX™ Green–negative, whereas SYTOX™ Green–positive events were considered non-viable (Supplementary Figure S1). For the purity assessment, neutrophils were defined by their characteristic immunophenotype as MPO^+^CD16^hi^CD14^lo^ cells, enabling the exclusion of monocytes and other contaminating leukocyte populations (Supplementary Figure S1). In all experimental assays, neutrophil viability was maintained at >99% and purity was >98% (Supplementary Figure S1).

### 2.7. Immunofluorescence-Based Study

Skin (lesional and normal skin) biopsies were fixed in 4% paraformaldehyde and dehydrated under an increasing concentration of ethanol, followed by paraffin embedding. Sections of 4 µm thickness were placed onto poly-L-lysine coated slides (Sigma Aldrich, St. Louis, MO, USA). Deparaffinized sections were rehydrated in a decreasing concentration of ethanol followed by washing in 1× PBS and subsequent antigen-retrieval in a sodium citrate buffer (pH 6) at 95 °C for 20 min. Processed skin tissue sections and stimulated neutrophils (on coverslip) were washed in 1× PBS and permeabilized using 0.2% Triton-X (Bio-Rad, Hercules, CA, USA), then blocked with 5% bovine serum albumin (BSA; HiMedia Laboratories, Mumbai, India) for 45 min in RT. After blocking, the sections/cells were subjected to incubation with primary antibody human anti-MPO (mouse polyclonal, 1:100, Thermo Fisher Scientific, Carlsbad, CA, USA) overnight at 4 °C. Cells/tissue sections were washed with 1×PBS having 0.025% Tween-20, followed by incubation with secondary antibody, i.e., FITC-conjugated goat anti-mouse (1:1000, Abcam, Cambridge, UK) for 1 h. Tissue sections/cells were then washed, followed by incubation with 4,6-diamidino-2-phenylindole (DAPI) (Sigma Aldrich, St. Louis, MO, USA) to counterstain the nuclei. To avoid photobleaching, VECTASHIELD Antifade Mounting Medium (Vector Laboratories, Newark, CA, USA) was used for mounting the glass slides using coverslips. A Nikon Ts2 Eclipse fluorescence microscope (Nikon, Long Beach, CA, USA) was used to acquire images. NETotic cells were quantified using immunofluorescence by demonstrating the colocalization of extracellular DNA (DAPI blue staining) with MPO (green), a defining feature of NET formation. DAPI+ intact nuclei were counted, and NET formation was expressed as the proportion of cells displaying NETotic morphology relative to the total number of seeded neutrophils, i.e., percentage of NETs = ((number of NETs/total number of neutrophils) × 100). Fluorescence intensity measurements were normalized to the number of DAPI+ neutrophils per microscopic field to correct for variations in cell density. Acquired images were processed using ImageJ (Fiji) software (version 1.54g) to ensure standardized quantification across all experimental conditions. Briefly, individual fluorescence channels were separated and converted to 8-bit grayscale images, followed by background subtraction. An automatic thresholding algorithm was applied to distinguish specific signal from background, and binary images were generated. NET structures were identified based on the characteristic features of decondensed and expanded DNA morphology, in combination with extracellular localization and colocalization with MPO. To exclude small debris and non-specific staining, an area threshold was applied during particle analysis, and only objects exceeding the predefined minimum area were included for quantification. NET formation was expressed as the percentage of NETotic cells relative to the total number of cells per field. For each sample, three independent microscopic fields were analyzed and averaged to minimize field-to-field variability.

### 2.8. Statistical Analysis

All statistical comparisons, including the graphs, were obtained using GraphPad Prism 5 (GraphPad Software, Inc., San Diego, CA, USA). Data distribution was assessed for normality using the D’Agostino–Pearson omnibus test. For normally distributed (parametric) datasets, comparisons between two groups were conducted using an unpaired two-tailed Student’s *t*-test (bar graph showing mean ± SEM) and for non-normally distributed (non-parametric) datasets, the Mann–Whitney U test was applied (represented as box/scatter plot showing the median (minimum–maximum)). For experiments involving more than two groups, one-way ANOVA (for normally distributed data) and the Kruskal–Wallis test (for non-parametric data) were used. Correlation analyses were performed using Pearson’s correlation analysis for normally distributed variables (Pearson correlation coefficient is represented as ‘r’ in the scatter plot). To minimize false-positive findings arising from multiple testing, Bonferroni correction was applied wherever applicable. A *p*-value of less than 0.05 (*p* < 0.05) was set as statistically significant in all the measurements.

## 3. Result

**Figure 1:**
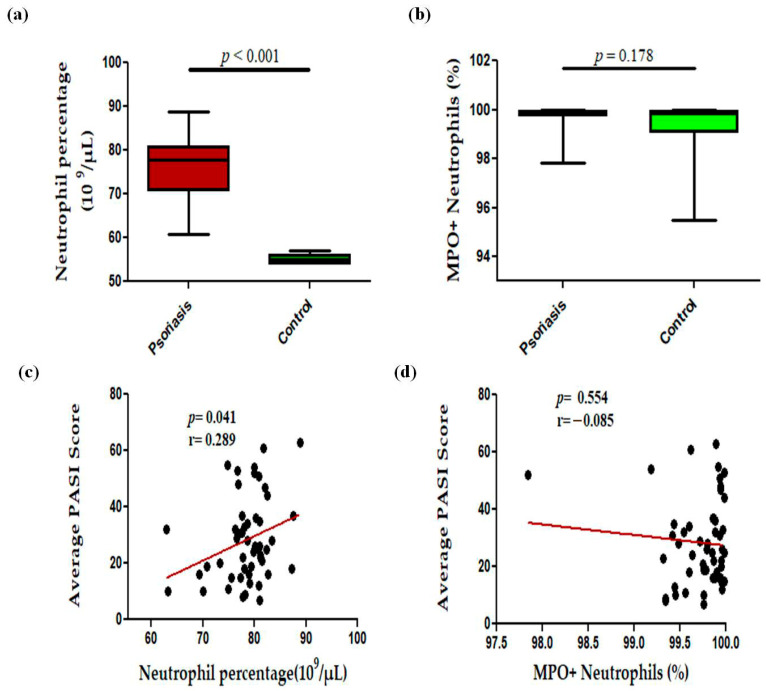
Comparison of percentage of (**a**) neutrophils (10^9^ per μL of blood), (**b**) MPO+ neutrophils in active psoriasis patients (*n* = 56) and controls (*n* = 56), correlation of (**c**) neutrophil percentage and PASI score, (**d**) percentage of MPO+ neutrophils and PASI score. Box-plots in panels (**a**,**b**) represent the median (minimum–maximum). Normality of the variables was assessed using the D’Agostino–Pearson omnibus test ((**a**,**b**) had not passed normality test and a Mann–Whitney U test was performed; (**c**,**d**) passed normality test and a Pearson’s correlation test was performed); r = Pearson correlation coefficient; *p* value was set at <0.05 significance.

**Figure 2:**
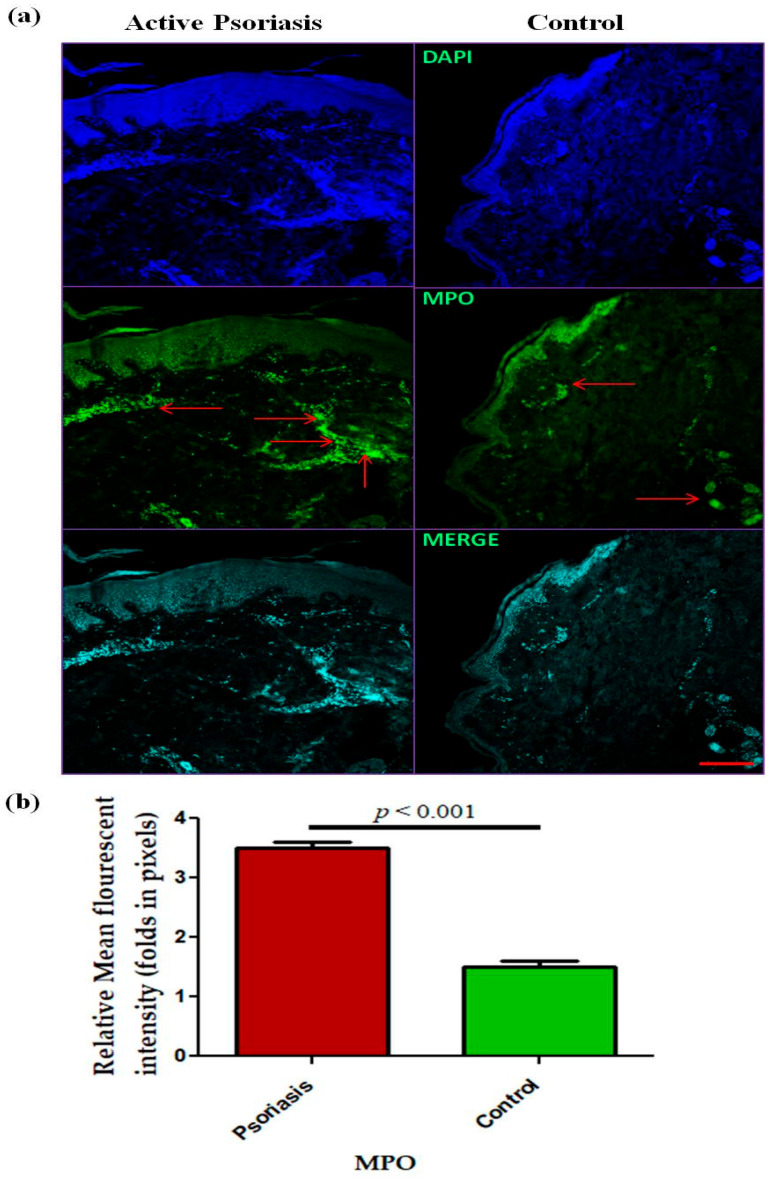
Immunofluorescence-based (representative images) localization of (**a**) MPO in lesional and control skin and (**b**) quantification (relative mean fluorescent intensity) of MPO in active psoriasis patients (*n* = 56) and controls (*n* = 56). MFI values were calculated from three independent fields per sample and averaged for each participant. Representative images (10× magnification) were shown (MPO = green, and nucleus = blue (DAPI)). Normality of the data was assessed using the D’Agostino–Pearson omnibus test. Since the data followed a normal distribution, statistical differences between groups were determined using a two-tailed unpaired Student’s *t*-test, (**b**). Bar graphs represent the mean ± SEM. Scale bar = 10 µm, *p* value was set at <0.05 significance. The red arrow indicated MPO staining.

In this study, we observed a significant (*p* < 0.001) increase in the levels of circulating neutrophils in active psoriasis patients compared to controls (Figure 1a). However, no significant difference was found in the percentage of MPO+ neutrophils between active psoriasis patients and controls (Figure 1b) (*p* = 0.178). A correlation analysis was performed, using Pearson’s correlation analysis, which demonstrated a marked positive correlation between the neutrophil frequency and the PASI score (r = 0.289, *p* = 0.041) (Figure 1c). However, the MPO+ neutrophil frequency showed no significant correlation with the PASI score (r = –0.085, *p* = 0.554) (Figure 1d). An immunofluorescence analysis revealed the localization of the MPO protein in skin tissues where marked MPO expression was observed in the lesional skin of active psoriasis patients compared to controls. A quantitative analysis of the mean fluorescence intensity (MFI) confirmed these observations where MPO exhibited significantly higher MFI values in the lesional skin of active psoriasis patients compared to controls (*p* < 0.001) (Figure 2).

**Figure 3:**
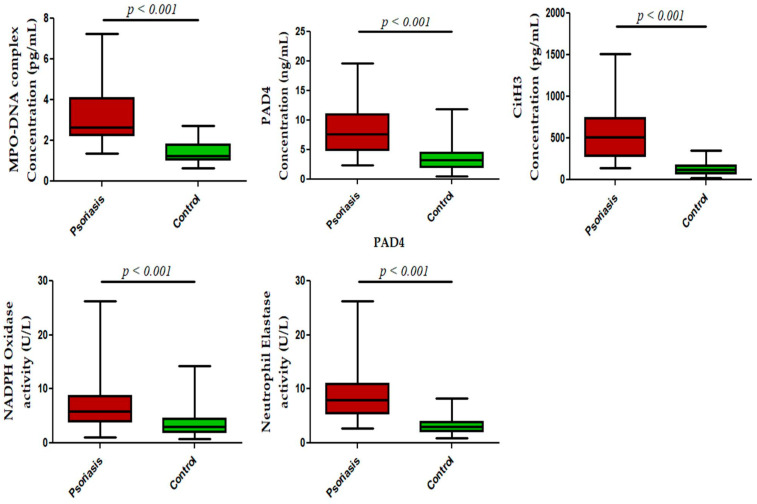
Status of NET-associated markers in the peripheral blood of active psoriasis patients (*n* = 56) and controls (*n* = 56). The y-axis represents analyte concentrations or enzyme activity. All ELISA measurements were performed in triplicate. Data are presented as the median (minimum–maximum). Normality was evaluated using the D’Agostino–Pearson omnibus test, followed by a Mann–Whitney U test (*p* value was set at <0.05 significance) for non-parametric data analysis.

**Figure 4:**
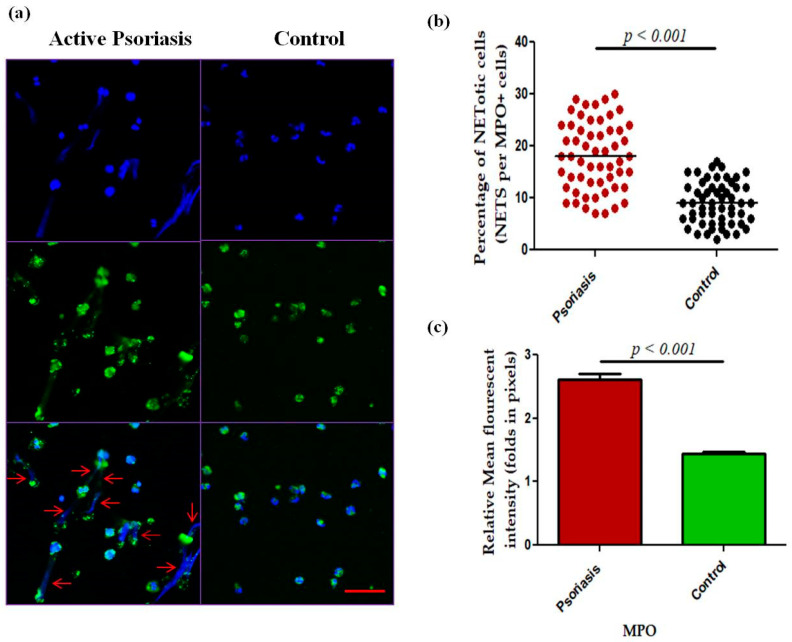
Comparison of (**a**) immunofluorescence (representative) images of the in vitro NET formation (PMA stimulated) potential of MPO+ neutrophils, (**b**) quantification of the percentage of NETotic cells, and (**c**) quantification (MFI) of MPO–DAPI in active psoriasis patients (*n* = 56) and controls (*n* = 56). Images were acquired using immunofluorescence microscopy at 40× magnification, with identical laser power, gain, and exposure settings applied to all samples. Data are presented as (**b**) the median (minimum–maximum), Mann–Whitney U test, or (**c**) the mean ± SEM (two-tailed unpaired Student’s *t*-test). Normality of all quantitative variables was assessed using the D’Agostino–Pearson omnibus test. Scale bar = 50 µm, MPO (green); DNA/nuclei (blue), (*p* value was set at <0.05 significance). The red arrow indicates MPO-DNA staining.

We next measured NET-related proteins and enzyme activities in the peripheral blood of active psoriasis patients and controls. We observed significantly increased levels of MPO–DNA complexes (*p* < 0.001), PAD4 (*p* < 0.001), and CitH3 (*p* < 0.001) in the serum of active psoriasis patients compared to controls reflecting elevated NET formation (Figure 3). Further, NET-associated enzyme activities, including NE (*p* < 0.001) and NADPH oxidase (*p* < 0.001), were significantly elevated in active psoriasis patients compared to controls (Figure 3). All ELISA assays were performed in triplicate, and the box-plot graphs (Mann–Whitney U test) represent the median (minimum–maximum). Next, we determined the NET-forming capacity and found a significant increase (*p* < 0.001) in the NETotic cells (per MPO+ neutrophils) in active psoriasis patients compared to controls (Figure 4), which was supported by mean fluorescence quantification (*p* < 0.001).

**Figure 5:**
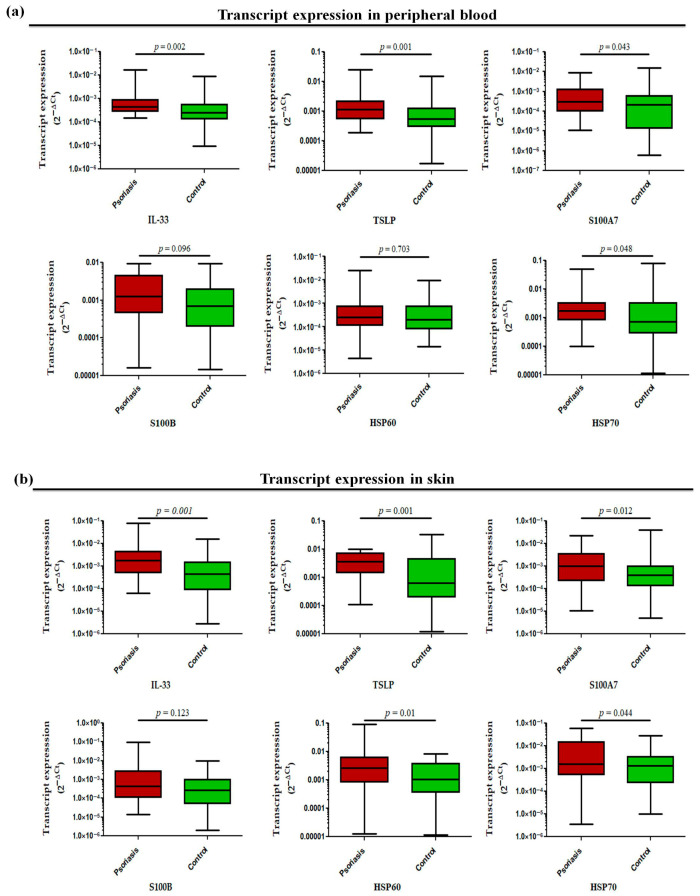
Relative transcript expression of alarmins (i.e., IL-33, TSLP, S100A7, HSP60, HSP70, and S100B) in (**a**) the peripheral blood and (**b**) the skin (lesional vs. normal skin) of active psoriasis patients (*n* = 56) and controls (*n* = 56). Gene expression was quantified using real-time qPCR, normalized to β-actin. The y-axis represents transcript expression (2^−ΔCt^ values). All qPCR reactions were performed in triplicate, and values from triplicates were averaged for each participant. Normality of all datasets was evaluated using the D’Agostino–Pearson omnibus test followed by a Mann–Whitney U test. Box-plots represent the median (minimum–maximum), and the *p* value was set at <0.05 significance.

The transcript profiling of alarmin genes in peripheral whole blood revealed significantly increased expression of all studied alarmins, such as IL-33 (*p* = 0.002), TSLP (*p* = 0.001), S100A7 (*p* = 0.043), and HSP70 (*p* = 0.048) except for S100B (*p* = 0.096) and HSP60 (*p* = 0.703), in active psoriasis patients compared to controls (Figure 5a). However, the corresponding transcript expression in the skin compartment revealed elevated expression of IL-33 (*p* = 0.001), TSLP (*p* = 0.001), S100A7 (*p* = 0.012), HSP60 (*p* = 0.01), and HSP70 (*p* = 0.044) in lesional psoriatic skin compared with normal skin, but no significant difference was observed for S100B in both groups (Figure 5b). The alarmins IL-33 and TSLP consistently demonstrated elevated expression in both skin and blood compartments. Considering the critical role of alarmins in psoriasis, we next determined the role of the two target alarmins, IL-33 and TSLP, in NET formation and their subsequent role in cytokine production and oxidative stress.

**Figure 6:**
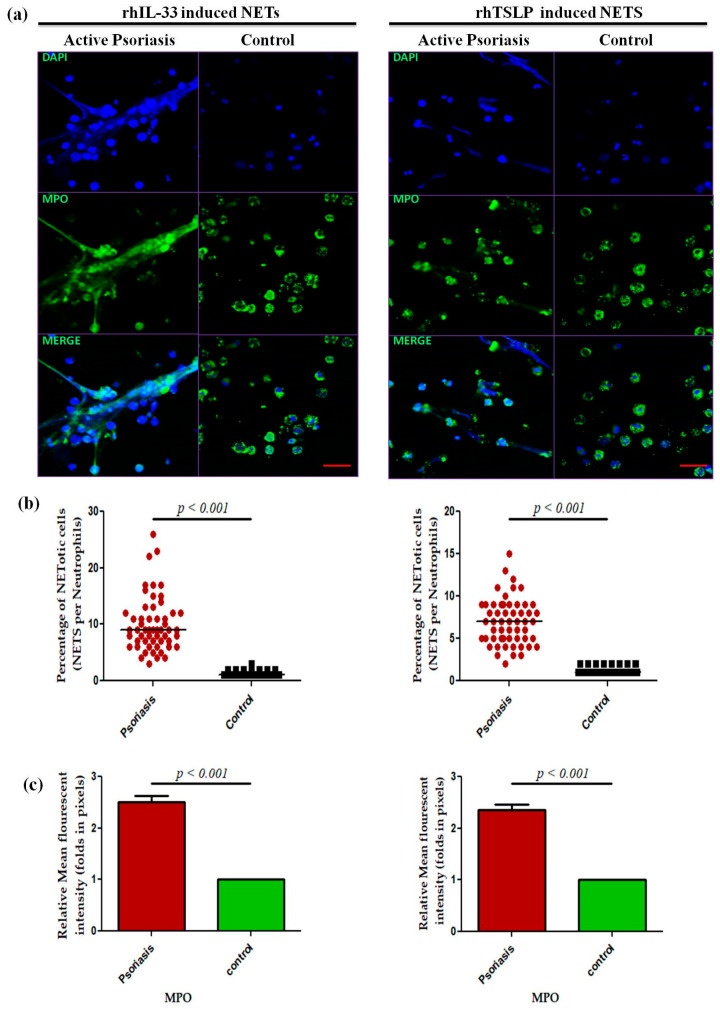
Comparison of the NET formation potential of MPO+ neutrophils stimulated with (**a**) immunofluorescence (representative) images of the in vitro NET formation (rhIL-33-induced and rhTSLP-induced) potential of MPO+ neutrophils, (**b**) quantification of the percentage of NETotic cells, and (**c**) quantification (MFI) of MPO–DAPI in active psoriasis patients (*n* = 56) and controls (*n* = 56). Imaging was performed at 40× magnification using identical laser power, gain, and exposure settings for all samples (MPO = green; nucleus = blue (DAPI)). Data are presented as (**b**) the median (minimum–maximum), Mann–Whitney U test, or (**c**) the mean ± SEM (two-tailed unpaired Student’s *t*-test). Normality of all quantitative variables was assessed using the D’Agostino–Pearson omnibus test. Scale bar = 50 µm, MPO (green); DNA/nuclei (blue), (*p* value was set at <0.05 significance).

A dose-dependent assay was performed for IL-33 and TSLP at 0 ng/m L (media only), 50 ng/mL, and 100 ng/mL for 4 h for the psoriasis and control groups that revealed a maximum response at 100 ng/mL (Supplementary Figure S2), and subsequent experiments were performed at the 100 ng/mL concentration. Isolated neutrophils from active psoriasis patients exhibited increased NET formation abilities upon stimulation with either rhIL-33 or rhTSLP compared to controls (Figure 6a), and it is further supported by the quantification data (*p* < 0.001) (Figure 6b,c). Similarly, the MPO mean fluorescence intensity (MFI) was significantly elevated in the IL-33- and TSLP-induced NETs of psoriasis patients compared to controls.

**Figure 7:**
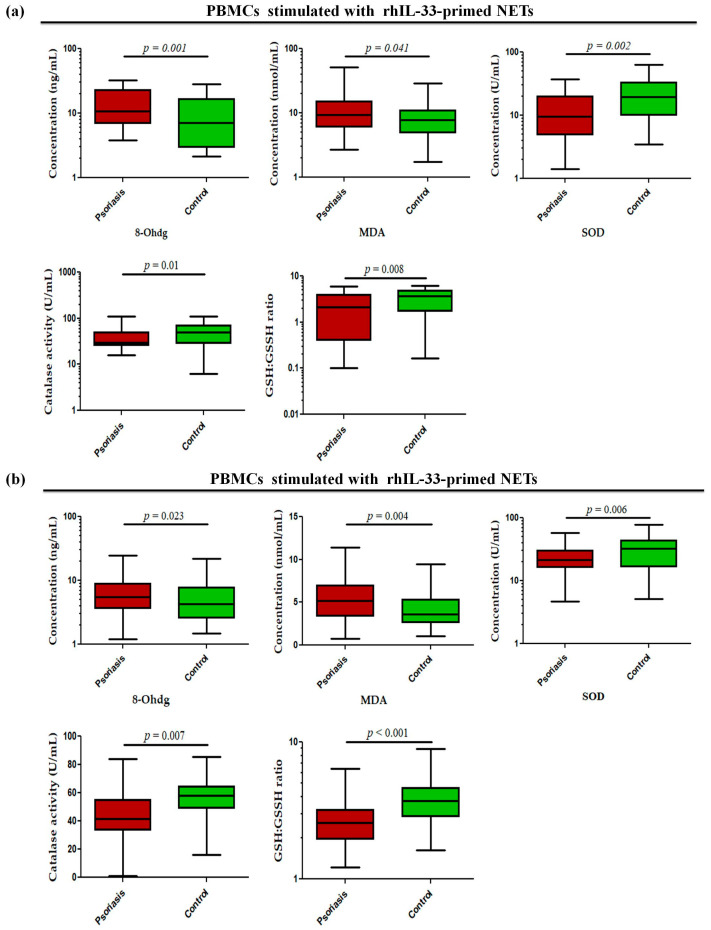
Status of oxidative stress markers (8-OHdG (ng/mL), MDA (nmol/mL), SOD activity (U/mL), catalase activity (U/mL), and GSH/GSSG ratio) in PBMCs stimulated with (**a**) rhIL-33-primed NETs and (**b**) rhTSLP-primed NETs of active psoriasis patients (*n* = 56) and controls (*n* = 56). The y-axis represents the analyte concentrations or enzyme activity. All ELISA measurements were performed in triplicate and averaged values were used for analysis. Data are presented as the median (minimum–maximum). Normality was evaluated using the D’Agostino–Pearson omnibus test, followed by a Mann–Whitney U test (*p* value was set at <0.05 significance) for non-parametric data analysis.

Since these alarmins are known to be secreted by keratinocytes, they may act as possible triggers in the skin to induce NETs and may further exacerbate inflammatory responses in the skin. To determine whether alarmin-primed NETs can modulate oxidative stress markers, such as oxidative DNA damage (8-OHdG), lipid peroxidation (MDA), GSH/GSSG ratio, and antioxidant enzyme activity (SOD and catalase) in psoriasis, the status of these markers was determined in PBMCs stimulated with rhIL-33- and rhTSLP-primed NETs. The PBMCs stimulated with rhIL-33-primed NETs showed significantly increased levels of 8-OHdG (*p* = 0.001) and MDA (*p* = 0.041) in active psoriasis patients compared to controls (Figure 7a). Further, the antioxidant capacity was reduced in active psoriasis patients, as reflected by lower SOD activity (*p* = 0.002), catalase activity (*p* = 0.01), and GSH/GSSG ratio (*p* = 0.008) compared to controls (Figure 7a). A similar trend was observed for PBMCs stimulated with rhTSLP-primed NETs, where significantly higher levels of 8-OHdG (*p* = 0.023) and MDA (*p* = 0.004) were observed along with substantial reductions in SOD (*p* = 0.006) and catalase activity (*p* = 0.007) and along with a lower GSH/GSSG ratio (*p* < 0.001) in active psoriasis patients compared to controls (Figure 7b). Additionally we also determined the direct effect of IL-33 and TSLP on oxidative stress markers. and observed a similar effect for rhIL-33- and rhTSLP-primed NETs (Supplementary Figure S3).

**Figure 8:**
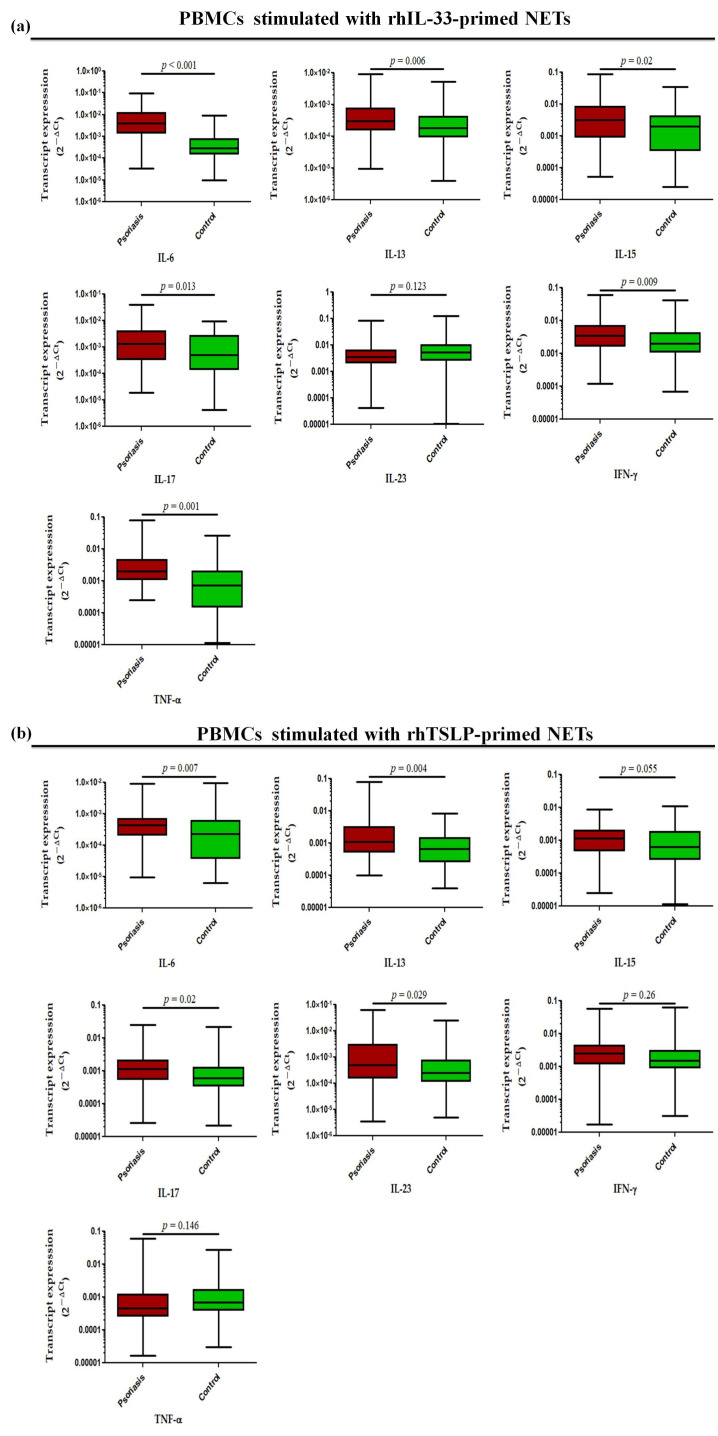
Relative mRNA expression levels of inflammatory cytokines (i.e., IL-6, IL-13, IL-17, IL-23, IFN-γ, and TNF-α) in PBMCs stimulated with (**a**) rhIL-33-primed NETs and (**b**) rhTSLP-primed NETs of active psoriasis patients (*n* = 56) and controls (*n* = 56). Gene expression was quantified using real-time qPCR, normalized to β-actin. The y-axis represents transcript expression (2^−ΔCt^ values). All qPCR reactions were performed in triplicate, and values from triplicates were averaged for each participant. Normality of all datasets was evaluated using the D’Agostino–Pearson omnibus test followed by a Mann–Whitney U test. Box-plots represent the median (minimum–maximum), and the *p* value was set at <0.05 significance).

Further, in a similar experimental set-up, we determined the status of pro-inflammatory cytokines, where transcript levels of IL-6 (*p* < 0.001), IL-13 (*p* = 0.006), IL-15 (*p* = 0.02), IL-17 (*p* = 0.013), TNF-α (*p* = 0.001), and IFN-γ (*p* = 0.009) were found to be significantly increased in the PBMCs treated with rhIL-33-primed NETs in active psoriasis patients compared to controls, but no significant difference was observed for IL-23 (Figure 8a). Similar trends were obtained for PBMCs challenged with rhTSLP-primed NETs in which the significantly elevated transcript levels of IL-6 (*p* = 0.007), IL-13 (*p* = 0.004), IL-15 (*p* = 0.055), IL-17 (*p* = 0.02), and IL-23 (*p* = 0.029) were observed in active psoriasis patients compared to controls (Figure 8b). However, no significant changes were found for the transcript levels of TNF-α and IFN-γ between the groups (Figure 8b). Additionally we also determined the direct effect of IL-33 and TSLP on the mRNA expression profile of pro-inflammatory cytokines, and observed similar trends for rhIL-33- and rhTSLP-primed NETs (Supplementary Figure S4).

## 4. Discussion

Psoriasis is a chronic inflammatory disease where adaptive immune components have been extensively investigated over past three decades. It is considered to be major health burden globally, as it encompasses not only dermatology (skin involvement) and rheumatology (joint involvement) but is associated with various metabolic and cardiovascular diseases [43,44,45,46,47]. The pathogenesis mainly revolves around pathogenic T cells that are influenced by genetic and environmental factors [45,47,48,49]. However, in recent years, the innate immune components have gained significant attention [46,47]. Psoriasis is now increasingly recognized as a disease initiated by epithelial injury caused by myriad of external/internal factors followed by innate immune activation that subsequently augments the adaptive immune responses. In consideration of that, our study was focused on studying the role of neutrophils in psoriasis with special emphasis on NETs, NET-associated markers, and alarmins as potent NET triggers, as well as the subsequent impact in driving inflammation and oxidative stress. Firstly, we determined the frequency of neutrophils in peripheral blood, and observed a marked increase in the percentage of neutrophils in active psoriasis patients with a positive correlation with disease severity. This data indicated a direct correlation of enhanced neutrophil frequency with disease progression. Previous studies have also reported the involvement of neutrophils in psoriasis, with increased neutrophil frequency in the peripheral blood and psoriasis skin [22,50,51,52] that is clinically correlated with disease severity supporting our findings. Recently, a neutrophil immunophenotyping-based study revealed the increased frequency of CD10+ and CD10− neutrophils in the peripheral blood of psoriasis patients. CD10+ neutrophils displayed distinct maturation stages with resemblance to aged neutrophils, and NETs formed by these neutrophils triggered the production of IFN-γ and IL-17 in T cells [22]. In consideration of that, we determined the frequency of circulating MPO+ neutrophils subsets, but no significant association was observed for MPO+ neutrophils with psoriasis, which may be due to the ubiquitous expression of MPO in both patient and control neutrophils. Therefore, understanding the neutrophil subsets in context with the NET formation potential is highly warranted.

Since MPO is predominantly expressed by activated neutrophils, we next localized the expression of the MPO protein in psoriatic lesional skin by immunofluorescence, where it was significantly increased in lesional skin compared with normal skin. This observation indicated the enhanced infiltration of MPO+ neutrophils and their subsequent activation in skin that may in turn augment the activation of other immune cells and inflammatory cascade. However, it has been reported that, under an inflammatory milieu, other immune cells, like macrophages and monocytes, can also express MPO, but it is largely limited to the brain/central nervous system [44]. Therefore, we gated MPO on monocytes, and no marked expression of MPO was observed on monocytes in active psoriasis patients and controls. MPO is located in the azurophilic granules of neutrophils accounting for almost 5% of its dry weight and is regarded as the most abundant neutrophil protein [45,46,47,48]. It plays a critical role in regulating the neutrophil function. In activated neutrophils, MPO is either translocated to phagosomes or it may be released extracellularly [45,46,47,48]. In infectious conditions, MPO elicits the production of ROS that aid in killing pathogens [49]. Owing to its strong negative charge due to arginine and lysine residues, MPO has the tendency to bind to nucleic acids (DNA/RNA), proteins (integrins), and lipids, and it can form a complex that can act as a potent immunogen [47,53,54,55]. The MPO–DNA complex has been widely reported in the pathogenesis of various autoimmune and inflammatory diseases, including psoriasis [56,57]. In the present study, we also found an increased concentration of the MPO–DNA complex in the serum of active psoriasis patients compared to controls. Recent studies have shown that neutrophils can form NETs, and MPO is critical in NET formation [45]. MPO works in coordination with NE and PAD4 to form NETs via decondensation of chromatins, and it is further aided by the citrullination of histones. The PAD4 is often activated by intracellular calcium and ROS. Increased ROS is associated with elevated levels of NADPH oxidase enzyme activity. The MPO and NE amplify the chromatin decondensation process that culminates in nuclear membrane rupture, leading to the release of decondensed chromatin. In consideration of that, we determined the NET-associated markers, namely CitH3, PAD4, NE, and NADPH oxidase. All these markers were significantly elevated in the peripheral blood of active psoriasis patients, indicating an exacerbated NET induction that may lead to the release of NET components in serum. Since these NET components are highly inflammatory by nature, systemic activation of the target immune cells is highly anticipated to further aggravate the progression of the disease.

Multiple factors can trigger NET formation, including a group of small endogenous molecules (danger signals) known as ‘alarmins’. Alarmins are released from apoptotic or necroptotic cells, and are recognized as potent triggers of NETs [58,59,60]. As mentioned above, MPO is a highly inflammatory molecule, and secreted MPO has the tendency to bind to the surface of endothelial and epithelial cells via interaction with glycosaminoglycans that are eventually internalized by exposed cells [59]. A similar mechanism is anticipated for keratinocytes, where MPO released from NETs can bind to the keratinocytes of lesional skin and manifest an elevated oxidative stress causing necroptosis [60], leading to the secretion of inflammatory alarmin molecules [51]. We identified a set of target alarmin molecules (i.e., IL-33, TSLP, S100A7, S100B, HSP 60 and HSP70), and observed a dysregulated mRNA expression pattern of these molecules in the peripheral blood and lesional skin of active psoriasis patients. However, our study has not described the cell-specific source of these alarmins, whether keratinocytes or immune cells (T cells, macrophages, or DCs).

Among the studied alarmins, we targeted IL-33 and TSLP, which showed consistent marked expression in peripheral blood. The precise role of IL-33 is not defined in psoriasis. Previous studies have reported the increased expression of IL-33 in serum and psoriatic lesions [61,62]. IL-33 inhibited IL-17 expression in the isolated CD4+ T cells of psoriasis patients. In a mouse model of psoriasis, IL-33 has been reported to inhibit the CD4+ T cells, the CD8+ T cells, and suppress the differentiation of Th17, thereby reducing the inflammation that is indicative of the IL-33-mediated protective function. However, in our study IL-33/IL-33-primed NETs showed an increased expression of inflammatory cytokines along with oxidative stress. Further, contradictory studies have reported increased IL-33 in the serum of psoriasis patients that is positively associated with the PASI score [63]. An increased frequency of mast cells has been demonstrated in psoriasis patients and in an IMQ-induced psoriasis mouse model. IL-33 activates the mast cells in an IMQ-induced psoriasis mouse model, thereby exacerbating the mast cell-mediated inflammation in psoriasis [64]. All these observations suggest that the complex role of IL-33 and the context-dependent dual roles, pro-inflammatory and anti-inflammatory, cannot be ruled out in psoriasis. The pathogenic role of TSLP has been widely reported in psoriasis, with increased serum TSLP that is correlated with the PASI score [65,66,67]. TSLP promotes Th17 cell differentiation by activating the DCs through the JAK/SYK pathway in a mouse model of psoriasis [68]. Further, downregulation of c-JUN and JUNB was identified in the bulge hair follicle stem cells (HF-SCs) of scalp psoriasis patients, and the inducible deletion of c-JUN and JUNB in HF-SCs of mouse model of psoriasis increased the skin inflammation and epidermal hyperplasia [69]. This effect is mediated by TSLP, and blocking TSLP mitigates inflammation and epidermal hyperplasia via reducing the STAT5 activation and expression of VEGFα in a psoriasis mouse model [69]. However, the role of IL-33 and TSLP as one of the triggers for NET formation in psoriasis has yet to be comprehensively defined. Therefore, we applied a hypothesis-free inductive approach to define the impact of IL-33 and TSLP in regulating NET formation in psoriasis and control neutrophils. Both IL-33 and TSLP were demonstrated to significantly increase NET formation in the isolated neutrophils of active psoriasis patients compared to controls, indicating the role of these alarmins as potential triggers of NETs. It will be interesting to target these molecules in alleviating the inflammatory cascade and oxidative stress in psoriasis, provided a functional validation in an appropriate experimental model. Inflammation and oxidative stress are the hallmark features of psoriasis, and there are substantial reports describing a myriad of effector molecules to mediate the same [33,36,70,71,72]. However, the role of IL-33 and TSLP in modulating the inflammation and oxidative stress in peripheral blood via NET aggravation has yet to be described. Therefore, we further extended our investigation to understand the role of these alarmin-induced NETs in eliciting oxidative stress and modulating the secretion of pro-inflammatory cytokines in the PBMCs of psoriasis. The IL-33- and TSLP-primed NETs revealed the disruption of redox balance in stimulated PBMCs, as indicated by upregulation of the markers of oxidative DNA damage and lipid peroxidation with a parallel decrease in ROS scavenging enzymes (i.e., SOD and catalase) and the GSH/GSSG ratio. However, similar effects were observed in the status of oxidative stress when PBMCs were directly challenged with IL-33 and TSLP, supporting the notion that IL-33 and TSLP are capable of inducing oxidative stress independently in the PBMCs of psoriasis patients.

Further, we observed that IL-33-primed NETs enhance the expression of IL-6, IL-13, IL-15, IL-17, IFN-γ, and TNF-α; whereas TSLP-primed NETs significantly induce the production of IL-6, IL-13, IL-17, and IL-23 pro-inflammatory cytokines in the PBMCs of psoriasis patients compared to controls. Similar effects were observed in the status of inflammatory cytokines when the PBMCs were directly challenged with IL-33 and TSLP, indicating their independent potential to upregulate the inflammatory responses. The role of all these cytokines is well documented in the pathogenesis of psoriasis. IL-6 secretion enhances STAT3 (signal transducer and activator of transcription 3) activation in keratinocytes and drives Th17 (T helper 17 cells) polarization, thereby amplifying the inflammatory signaling initiated by NET-derived stimuli [73]. Elevated serum IL-15 is reported in psoriasis, and it is known to trigger IL-17 secretion by T cells and to maintain memory Th17 cells [74,75]. IL-17 is produced predominantly by Th17 cells, γδ (gamma delta) T cells, neutrophils, and ILC3 (type 3 innate lymphoid cells) [76,77]; it is considered to be the critical driver of psoriasis, and NETs are known to induce Th17 cell differentiation in psoriasis. IL-23 is the master regulator of Th17 cell survival and differentiation. The present study demonstrated the elevated expression of IL-17 in PBMCs stimulated with IL-33-primed NETs and TSLP-primed NETs, but the marked expression of IL-23 reported in PBMC exposed to TSLP-primed NETs only. This observation supported the notion that the IL-33/TSLP-NETs axis is involved in driving systemic inflammation in psoriasis. Although classically linked to type-2 immunity, IL-13 is not extensively investigated in psoriasis but it known to act synergistically with other inflammatory cytokines, such as IL-17 and IFN-γ [78,79]. In our investigation, both of the alarmins, IL-33 and TSLP, upregulated the expression of IL-13 in PBMCs via alarmin–NET mediated induction. TNF-α and IFN-γ are extensively studied in psoriasis, and their upregulated expression is widely reported in the peripheral blood and lesion skin of psoriasis patients [80,81,82,83,84,85]. However, therapies targeting these effector cytokines have revealed limited efficacies in the management of psoriasis. In our investigation, IL-33-primed NETs significantly upregulated the expression of TNF-α and IFN-γ in PBMCs, but the same was not evident for TSLP-primed NETs. All these observation indicated the potential role of IL-33 and TSLP in inducing the activation of an inflammatory cascade via NETs in the peripheral blood that may be manifested as systemic inflammation, eventually leading to the development/progression of the inflammatory plaques in psoriasis.

### Limitations

The present study defined the role of the alarmins IL-33 and TSLP in triggering ex vivo NET formation in the neutrophils of active psoriasis vulgaris patients. The IL-33- and TSLP-primed NETs induce the oxidative stress and inflammatory responses in the PBMCs of psoriasis patients as the result of heightened immune activation and the production of effector cytokines. Our study addressed only systemic inflammation, and immune cell specific investigation is highly warranted. However, the role of IL-33- and TSLP-primed NETs in modulating keratinocyte proliferation and differentiation using robust in vitro or in vivo experimental model is necessary to develop a better understanding of these alarmins in regulating skin homeostasis in psoriasis.

## 5. Conclusions

In this study, we demonstrated the nexus of the alarmins–NETs–oxidative stress loop that is the key driver of inflammation in psoriasis pathogenesis. In psoriatic skin, alarmins (IL-33 and TSLP) are primarily secreted from damaged or stressed keratinocytes under the influence of intrinsic or extrinsic factors and partly contributed by activated immune cells. These alarmins bind to their cognate receptors present on neutrophils (and also to other effector immune cells) facilitating its activation and subsequent NET formation. The NET armamentarium is loaded with potential inflammatory molecules that can augment the activation of target effector T cells (CD8+ T cells, Th1/Th17, γδ T cells) and other immune cells (neutrophils, NK cells, macrophages, DCs). The activated immune cells further secrete the pro-inflammatory cytokines triggering the apoptosis/necroptosis of keratinocytes, which in turn promote the alarmin release. This vicious cycle leads to the generation of potent ROS that breaks down the redox balance and further intensifies the oxidative stress and inflammation in psoriasis. This enhanced oxidative stress and inflammatory cascade may disrupt the regulated proliferation of keratinocyte resulting in excessive keratinocyte proliferation turnover that is precipitated as the inflammatory plaque formation in psoriatic skin. In normal skin, homeostasis is maintained by a robust anti-inflammatory and redox state that is governed by regulated immune response. The findings of the present study and its implications in psoriasis pathogenesis is summarized by the schematic representation in Graphical Abstract. In conclusion, alarmins such as IL-33 and TSLP can be targeted in psoriasis to breakdown the NET-mediated vicious inflammatory loop orchestrated by neutrophils. However, the impact of these alarmins in regulating NETs and keratinocyte proliferation require further functional validation in a robust in vitro/in vivo experimental model.

## Figures and Tables

**Table 1:** Demographic description of the psoriasis patients and controls.

Clinical Variables	Active Psoriasis Patients (Psoriasis Vulgaris)	Control	*p* Value
Total Participants	N = 56	N = 56	
Male	N = 47	N = 39	
Female	N = 9	N = 17	
Age (Mean ± SD) (in years)			
Total number of subjects	36.89 ± 12.73	33.51 ± 8.14	
Male	36.71 ± 13.58	33.62 ± 8.03	
Female	37.27 ± 11.21	33.36 ± 8.29	
PASI score (Mean ± SD)	37.27 ± 14.11	-	
Psoriasis (Mild) (PASI < 10)	N = 5	-	
Psoriasis (Moderate to severe) (PASI ≥ 10)	N = 51	-	
Total disease duration (in years) (Mean ± SD)	9.80 ± 6.51	-	
BMI (Mean ± SD)	23.4 ± 1.2	22.9 ± 1.1	*p* > 0.05
Blood Pressure			*p* > 0.05
Systolic blood pressure (SBP)			
(normal range: <120 mmHg)			
Range	110–118 mmHg	108–114 mmHg	
Mean ± SD	116.6 ± 3.0 mmHg	113.5 ± 2.8 mmHg	
Diastolic blood pressure (DBP)			
(normal range: <80 mmHg)			
Range	70–78 mmHg	68–76 mmHg	
Mean ± SD	74.6 ± 2.3 mmHg	72.4 ± 2.2 mmHg	
Fasting sugar glucose			*p* > 0.05
(normal range: 70–99 mg/dL)			
Range	88–98 mg/dL	82–90 mg/dL	
Mean ± SD	93.3 ± 2.7 mg/dL	86.4 ± 2.4 mg/dL	
HbA1c			*p* > 0.05
(normal range:			
Normal: < 5.7%			
Prediabetes: 5.7–6.4%			
Diabetes: ≥ 6.5%)			
Range	4.8–5.4%	4.7–5.1%	
Mean ± SD	5.42 ± 0.32	5.28 ± 0.30	
Thyroid profile test			*p* > 0.05
T3			
(normal range: 80–200 ng/dL)			
Range	112–125 ng/dL	105–115 ng/dL	
Mean ± SD	119.1 ± 3.3 ng/dL	110.4 ± 2.9 ng/dL	
T4			
(normal range: 5.1–14.1 µg/dL)			
Range	7.6–8.7 µg/dL	7.1–8.0 µg/dL	
Mean ± SD	8.18 ± 0.28 µg/dL	7.55 ± 0.26 µg/dL	
TSH			
(normal range: 0.27–4.20 µIU/mL)			
Range	1.8–2.6 µIU/mL	1.4–2.2 µIU/mL	
Mean ± SD	2.18 ± 0.19 µIU/mL	1.78 ± 0.20 µIU/mL	
Vitamin D			*p* > 0.05
(normal range: 30–50 ng/mL)			
Range	26–48	28–49	
Mean ± SD	34.8 ± 6.2	36.5 ± 5.8	
B12			*p* > 0.05
(normal range: 197–771 pg/mL)			
Range	260–620	280–650	
Mean ± SD	412 ± 96	438 ± 102	
Homocysteine			*p* > 0.05
(normal range: 5–15 µmol/L)			
Range	6.5–14.8	6.2–14.2	
Mean ± SD	10.8 ± 2.4	10.1 ± 2.2	
Hs CRP			*p* > 0.05
(normal range: 1.0–5.0 mg/L)			
Range	1.1–3.9	0.34–0.46	
Mean ± SD	2.48 ± 0.86	0.40 ± 0.03	
CK-MB			*p* > 0.05
(normal range: 0–25 U/L)			
Range	9–24	8–22	
Mean ± SD	16.8 ± 4.2	15.6 ± 3.8	
Trop T			*p* > 0.05
(normal range: <14 ng/L)			
Range	3.2–12.4	3.0–11.6	
Mean ± SD	7.6 ± 2.1	6.9 ± 1.9	
Metabolic/autoimmune/cardiovascular comorbidities (diabetes, hypertension, atherosclerosis, RA, IBD, SLE, etc.)	None	None	
Associated skin disease (vitiligo, AA, Acne, dermatitis, PsA, etc.)	None	None	
Smoking	None	None	
Alcohol or any drug addiction	None	None	
Treatment (Systemic/topical (e.g., corticosteroids, retinoids, phototherapy, methotrexate, cyclosporine, etc.))	None	None	

Abbreviations: BMI = body mass index; RA = rheumatoid arthritis; IBD = inflammatory bowel disease; SLE = systemic lupus erythematosus; PsA = psoriatic arthritis; TSH = Thyroid stimulating hormone; Hs CRP = high-sensitivity C-reactive protein; CK-MB = creatine phosphokinase-MB; Trop T = troponin *T* test; mg/dL = milligrams per deciliter; mIU/L = milli-international units per liter; ng/mL = nanograms per milliliter; pg/mL = picograms per milliliter; µmol/L = micromoles per liter; mg/L = milligrams per liter; U/L = units per liter; ng/L = nanograms per liter. All the variables are presented as mean ± SD (standard deviation). Normality test was performed for all parameters using the D’Agostino–Pearson omnibus test; an unpaired Student’s *t*-test was used for statistical comparison. A *p*-value of less than 0.05 (*p* < 0.05) was considered statistically significant.

## Data Availability

The original contributions presented in this study are included in the article/Supplementary Material. Further inquiries can be directed to the corresponding author.

## Supplementary Materials

The following supporting information can be downloaded at: https://www.mdpi.com/article/10.3390/antiox15010071/s1.
