# Supplementary material for: Enhanced Alarmin Secretion Exacerbates Neutrophil Extracellular Trap (NET) Formation in Active Psoriasis: Implication of IL-33 and TSLP in Driving NET Formation, Inflammation and Oxidative Stress in Psoriasis
Source: Antioxidants (Basel). 2026 Jan 6;15(1):71. doi: 10.3390/antiox15010071 (PMC12837554; doi:10.3390/antiox15010071)
Supplement: Supplementary file 1 [file antioxidants-15-00071-s001.zip › antioxidants-4039003-supplementary.pdf]

antioxidants

Supplementary Data

Figure S1. Flow cytometry based assessment of neutrophil purity and viability in active psoriasis patients and healthy control. (a) Representative flow cytometry based dot plots showing neutrophils (i.e. $MPO^{+}CD16^{hi}$ $CD14^{lo}$cells) based on CD16 and CD14 expression within the MPO^{+} gated population. (b) Quantitative comparison of neutrophil population expressed as the percentage of $MPO^{+}CD16^{hi}$ $CD14^{lo}$ cells between active psoriasis patients and healthy controls. (c) Representative flow cytometry dot plots showing the gating strategy used to identify viable neutrophil cells. (d) Quantitative comparison of neutrophil viability, expressed in percentage. Statistical analysis was performed using an unpaired Student's t-test (Data were presented as mean ± SEM). Note: No significant difference was observed in the purity (p = 0.297) and viability (p = 0.091) between the groups.

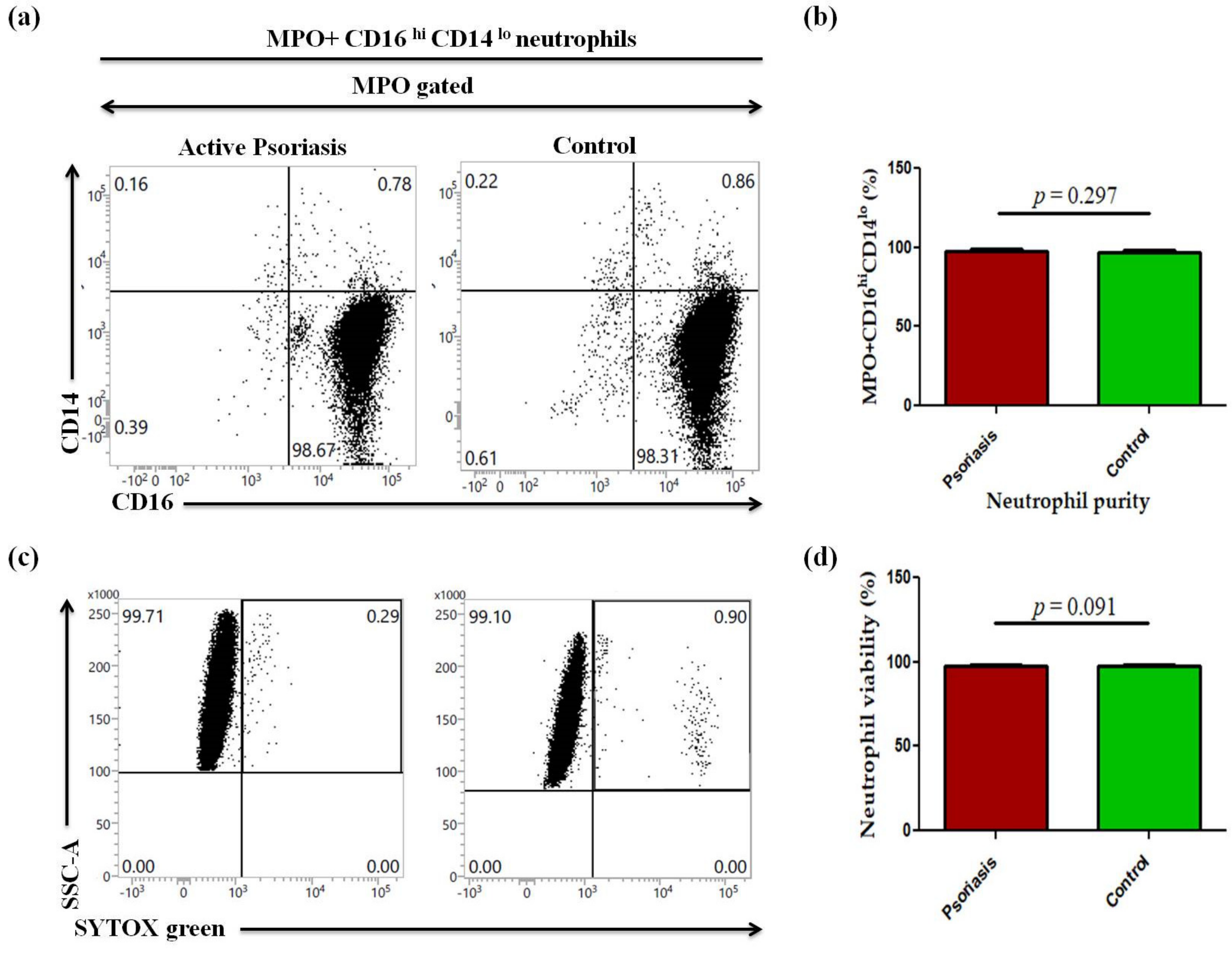

Figure S2. **Comparison of dose dependant effect of in rhIL-33 (S2(a)) and rhTSLP (S2(b)) on NET formation in active psoriasis patients (n=56) and healthy controls (n=56).** Upper panel: Representative immunofluorescence images of PBMCs stimulated with different doses of rhIL-33 and rhTSLP (i.e. 0 ng/mL (media only), 50 ng/mL, and 100 ng/mL). Lower panel: Quantification of NETotic cells and MFI for both the groups. Each dot represents an individual subject. Normality of all quantitative variables was assessed using the D'Agostino-Pearson Omnibus test followed by Kruskal-Wallis test based comparison. Scale bar = 50 µm, MPO (green); DNA/nuclei (blue), (p value was set at < 0.05 significance; where $p < 0.01$ (**), $p < 0.001$ (***), and *ns* = not significant).

S2 (a) **NETs stimulated with rhIL-33**

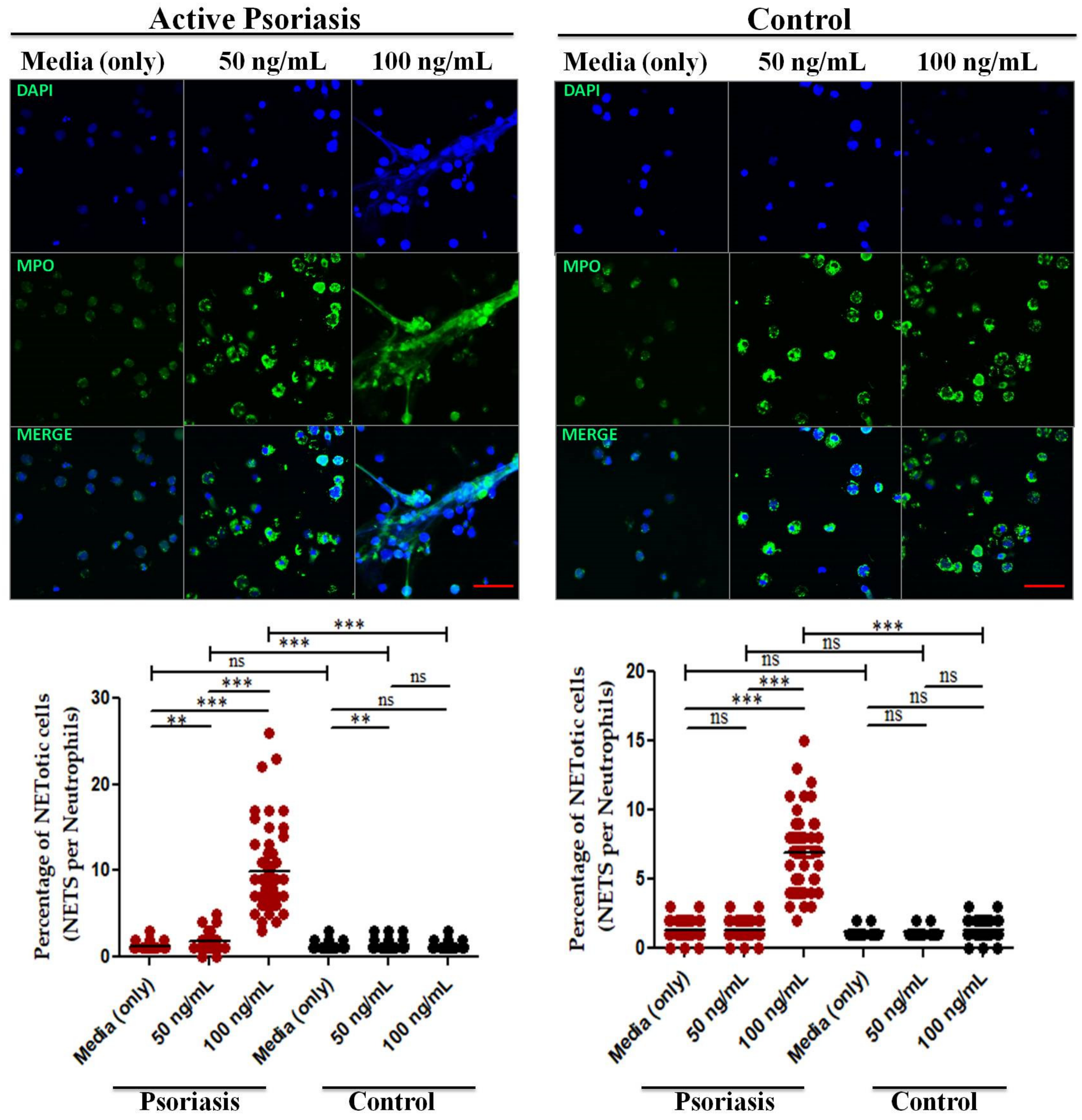

https://doi.org/10.3390/antiox15010071

S2 (b)

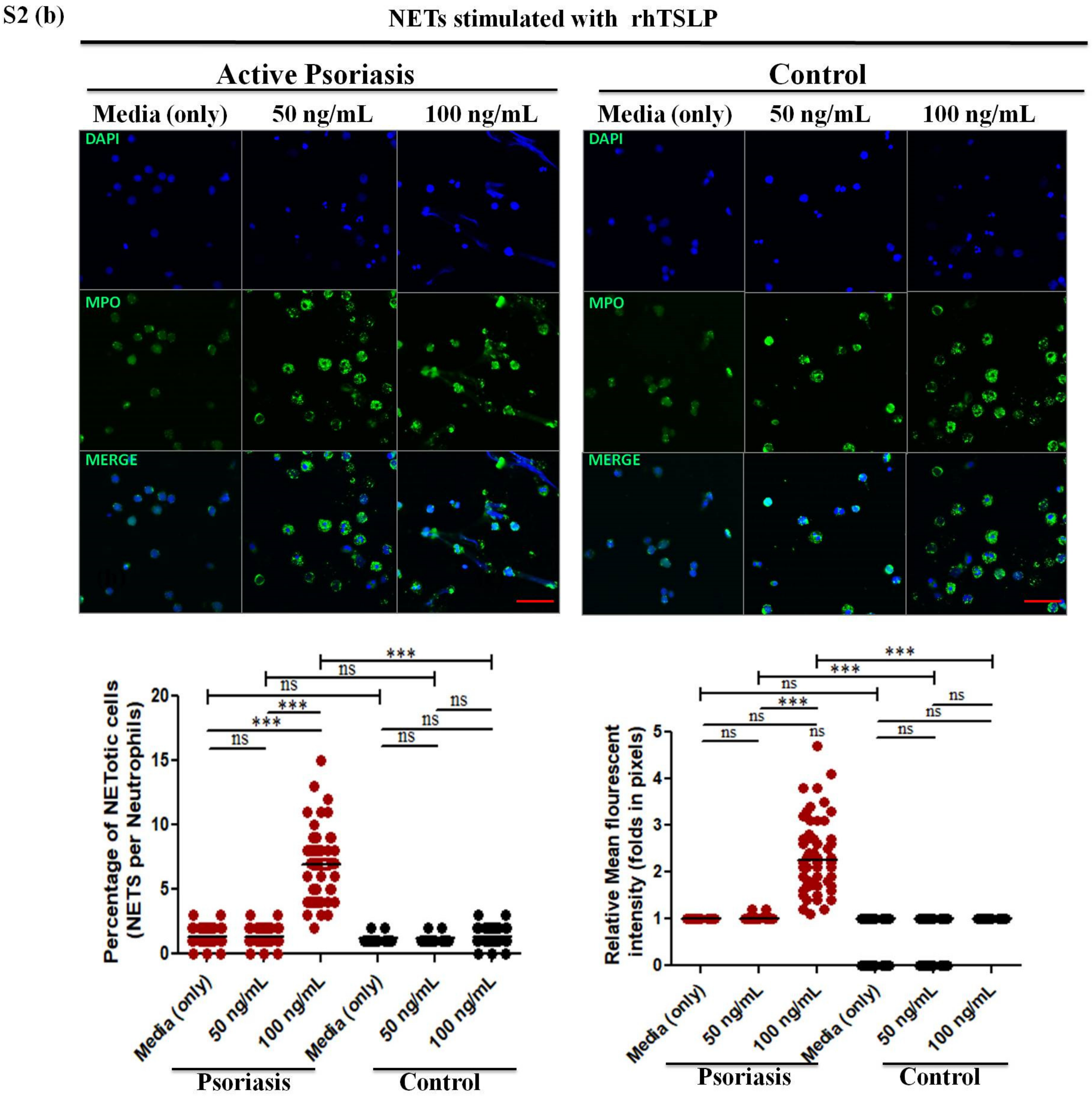

https://doi.org/10.3390/antiox15010071

Figure S3: **Status of oxidative stress markers (**8-OHdG (ng/mL), MDA (nmol/mL), SOD activity (U/mL), catalase activity (U/mL), and GSH/GSSG ratio) **in PBMCs stimulated with (a) rhIL-33 (b) rhTSLP of active psoriasis patients** (n = 56) **and controls** (n = 56)**.** The y-axis represented the analyte concentrations or enzyme activity. All ELISA measurements were performed in triplicate and averaged values were used for analysis. Data were presented as median (minimum-maximum). Normality was evaluated using the D'Agostino-Pearson Omnibus test, followed by Mann–Whitney U test (*p* value was set at < 0.05 significance) for non-parametric data analysis.

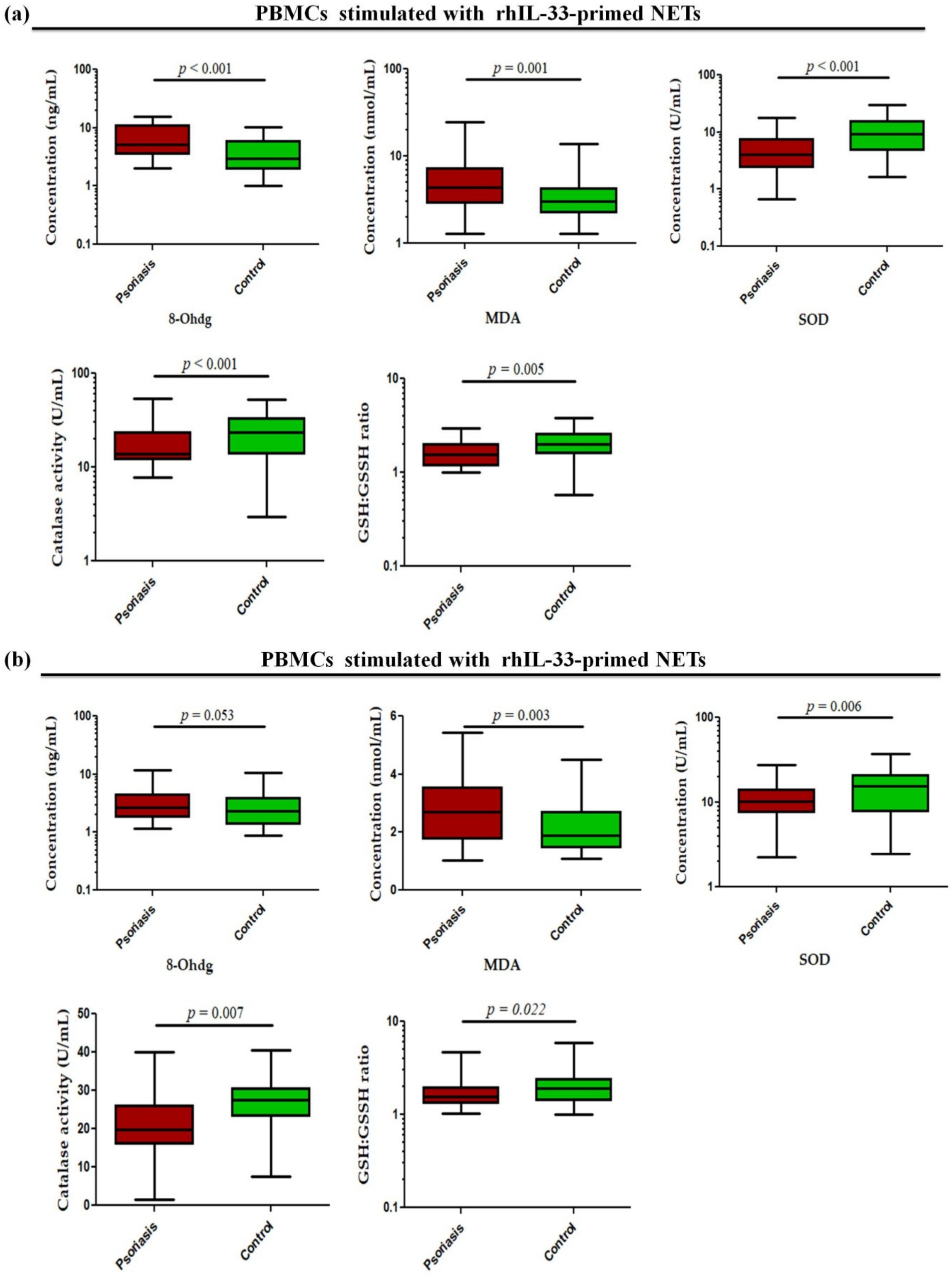

Figure S4. Relative mRNA expression levels of inflammatory cytokines (i.e. IL-6, IL-13, IL-17, IL-23, IFN-γ, and TNF-α) in PBMCs stimulated with (a) rhIL-33 (b) rhTSLP of active psoriasis patients (n = 56) **and controls** (n = 56)**.** Gene expression was quantified using real-time qPCR, normalized to β-actin. The y-axis represented transcript expression ($2^{-\Delta Ct}$ values). All qPCR reactions were performed in triplicate, and values from triplicates were averaged for each participant. Normality of all datasets was evaluated using the D'Agostino-Pearson Omnibus test followed by Mann–Whitney U test. Box-plot represented median (minimum-maximum), and *p* value was set at < 0.05 significance).

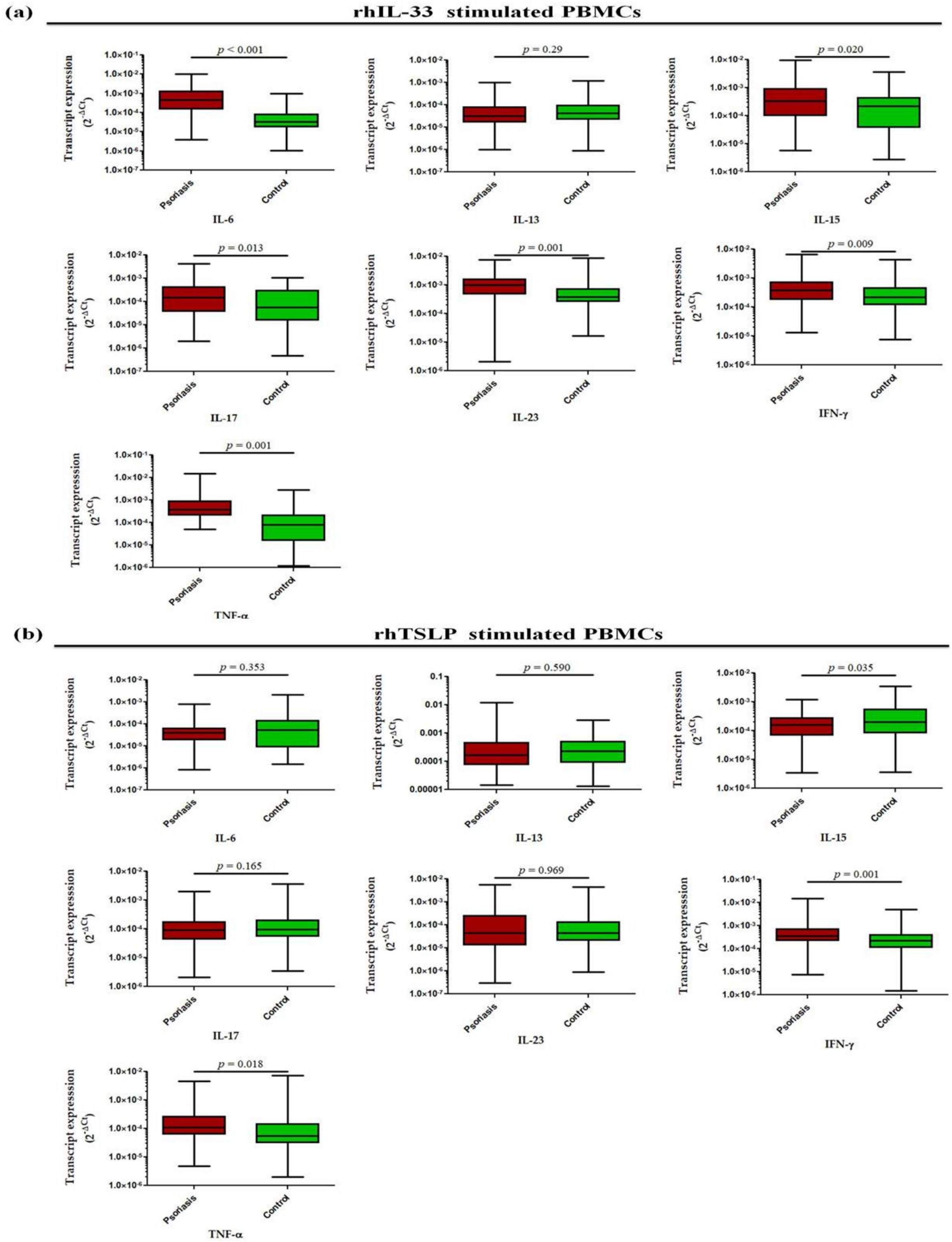

https://doi.org/10.3390/antiox15010071
